# Supplementary material for: Determining an optimal case definition using mid-upper arm circumference with or without weight for age to identify childhood wasting in the Philippines
Source: PLoS One. 2024 Dec 27;19(12):e0315253. doi: 10.1371/journal.pone.0315253 (PMC11676897; doi:10.1371/journal.pone.0315253)
Supplement: S1 File — (DOCX) [file pone.0315253.s001.docx]

Supplementary File 1 (S1)

Table 1. Diagnostic performance of MUAC cutoffs in identifying moderate wasting

| **MUAC Cut-off** | ***Cases (n)*** | ***Sensitivity*** | ***Specificity*** | ***PPV*** | ***NPV*** | ***Youden*** | ***AUROC*** | ***95% CI*** | |
| --- | --- | --- | --- | --- | --- | --- | --- | --- | --- |
| ***All sample*** |  |  |  |  |  |  |  | **LB** | **UB** |
| <10cm | 55 | 0.21% | 99.82% | 5.45% | 95.35% | 0.000 | 0.500 | 0.499 | 0.501 |
| <10.1cm | 60 | 0.28% | 99.81% | 6.67% | 95.35% | 0.001 | 0.500 | 0.499 | 0.502 |
| <10.2cm | 77 | 0.49% | 99.76% | 9.09% | 95.36% | 0.003 | 0.501 | 0.499 | 0.503 |
| <10.3cm | 84 | 0.49% | 99.74% | 8.33% | 95.36% | 0.002 | 0.501 | 0.499 | 0.503 |
| <10.4cm | 98 | 0.78% | 99.70% | 11.22% | 95.37% | 0.005 | 0.502 | 0.500 | 0.505 |
| <10.5cm | 102 | 0.78% | 99.69% | 10.78% | 95.37% | 0.005 | 0.502 | 0.500 | 0.505 |
| <10.6cm | 112 | 0.85% | 99.66% | 10.71% | 95.37% | 0.005 | 0.503 | 0.500 | 0.505 |
| <10.7cm | 131 | 1.27% | 99.61% | 13.74% | 95.39% | 0.009 | 0.504 | 0.501 | 0.507 |
| <10.8cm | 135 | 1.27% | 99.60% | 13.33% | 95.39% | 0.009 | 0.504 | 0.501 | 0.507 |
| <10.9cm | 160 | 1.97% | 99.55% | 17.50% | 95.42% | 0.015 | 0.508 | 0.504 | 0.511 |
| <11cm | 170 | 2.04% | 99.52% | 17.06% | 95.42% | 0.016 | 0.508 | 0.504 | 0.511 |
| <11.1cm | 196 | 2.61% | 99.45% | 18.88% | 95.44% | 0.021 | 0.510 | 0.506 | 0.514 |
| <11.2cm | 247 | 3.81% | 99.34% | 21.86% | 95.49% | 0.031 | 0.516 | 0.511 | 0.521 |
| <11.3cm | 278 | 4.44% | 99.26% | 22.66% | 95.52% | 0.037 | 0.519 | 0.513 | 0.524 |
| <11.4cm | 339 | 5.64% | 99.11% | 23.60% | 95.56% | 0.047 | 0.524 | 0.518 | 0.530 |
| <11.5cm | 366 | 6.06% | 99.04% | 23.50% | 95.58% | 0.051 | 0.525 | 0.519 | 0.532 |
| <11.6cm | 437 | 7.05% | 98.84% | 22.88% | 95.62% | 0.059 | 0.529 | 0.523 | 0.536 |
| <11.7cm | 519 | 8.67% | 98.64% | 23.70% | 95.68% | 0.073 | 0.537 | 0.529 | 0.544 |
| <11.8cm | 567 | 9.51% | 98.52% | 23.81% | 95.71% | 0.080 | 0.540 | 0.532 | 0.548 |
| <11.9cm | 704 | 11.84% | 98.16% | 23.86% | 95.80% | 0.100 | 0.550 | 0.542 | 0.558 |
| <12cm | 779 | 13.32% | 97.97% | 24.26% | 95.86% | 0.113 | 0.556 | 0.548 | 0.565 |
| <12.1cm | 985 | 16.49% | 97.42% | 23.76% | 95.99% | 0.139 | 0.570 | 0.560 | 0.579 |
| <12.2cm | 1318 | 20.44% | 96.47% | 22.00% | 96.13% | 0.169 | 0.585 | 0.574 | 0.595 |
| <12.3cm | 1470 | 22.27% | 96.03% | 21.50% | 96.20% | 0.183 | 0.592 | 0.581 | 0.602 |
| <12.4cm | 1838 | 26.29% | 94.97% | 20.29% | 96.35% | 0.213 | 0.606 | 0.595 | 0.618 |
| <12.5cm | 1992 | 27.77% | 94.51% | 19.78% | 96.41% | 0.223 | 0.611 | 0.600 | 0.623 |
| <12.6cm | 2233 | 30.51% | 93.82% | 19.39% | 96.51% | 0.243 | 0.622 | 0.610 | 0.634 |
| <12.7cm | 2638 | 35.02% | 92.64% | 18.84% | 96.69% | 0.277 | 0.638 | 0.626 | 0.651 |
| <12.8cm | 2818 | 36.93% | 92.12% | 18.59% | 96.77% | 0.290 | 0.645 | 0.633 | 0.658 |
| <12.9cm | 3310 | 41.09% | 90.63% | 17.61% | 96.93% | 0.317 | 0.659 | 0.646 | 0.671 |
| <13cm | 3611 | 44.47% | 89.76% | 17.47% | 97.07% | 0.342 | 0.671 | 0.658 | 0.684 |
| <13.1cm | 4209 | 49.47% | 87.95% | 16.68% | 97.28% | 0.374 | 0.687 | 0.674 | 0.700 |
| <13.2cm | 5181 | 55.53% | 84.91% | 15.21% | 97.51% | 0.404 | 0.702 | 0.689 | 0.715 |
| <13.3cm | 5620 | 58.35% | 83.53% | 14.73% | 97.63% | 0.419 | 0.709 | 0.696 | 0.722 |
| <13.4cm | 6450 | 62.93% | 80.91% | 13.84% | 97.81% | 0.438 | 0.719 | 0.706 | 0.732 |
| <13.5cm | 6807 | 64.76% | 79.77% | 13.50% | 97.89% | 0.445 | 0.723 | 0.710 | 0.735 |
| <13.6cm | 7414 | 67.79% | 77.83% | 12.98% | 98.02% | 0.456 | 0.728 | 0.716 | 0.741 |
| <13.7cm | 8328 | 72.02% | 74.90% | 12.27% | 98.21% | 0.469 | 0.735 | 0.723 | 0.747 |
| <13.8cm | 8803 | 74.14% | 73.37% | 11.95% | 98.31% | 0.475 | 0.738 | 0.726 | 0.749 |
| <13.9cm | 9807 | 77.94% | 70.10% | 11.28% | 98.49% | 0.480 | 0.740 | 0.729 | 0.751 |
| **<14cm** | **10305** | **79.63%** | **68.47%** | **10.97%** | **98.57%** | **0.481** | **0.741** | **0.730** | **0.751** |
| <14.1cm | 11289 | 82.10% | 65.21% | 10.32% | 98.68% | 0.473 | 0.737 | 0.726 | 0.747 |
| <14.2cm | 12716 | 85.55% | 60.48% | 9.55% | 98.85% | 0.460 | 0.730 | 0.721 | 0.740 |
| <14.3cm | 13319 | 86.33% | 58.44% | 9.20% | 98.87% | 0.448 | 0.724 | 0.714 | 0.733 |
| <14.4cm | 14393 | 88.30% | 54.85% | 8.71% | 98.97% | 0.432 | 0.716 | 0.707 | 0.725 |
| <14.5cm | 14820 | 88.94% | 53.41% | 8.52% | 99.00% | 0.423 | 0.712 | 0.703 | 0.720 |
| <14.6cm | 15567 | 90.20% | 50.91% | 8.22% | 99.07% | 0.411 | 0.706 | 0.697 | 0.714 |
| <14.7cm | 16688 | 91.47% | 47.12% | 7.78% | 99.13% | 0.386 | 0.693 | 0.685 | 0.701 |
| <14.8cm | 17215 | 92.11% | 45.34% | 7.59% | 99.16% | 0.374 | 0.687 | 0.680 | 0.695 |
| <14.9cm | 18349 | 93.59% | 41.51% | 7.24% | 99.25% | 0.351 | 0.676 | 0.669 | 0.682 |
| <10cm or WAZ <-2 | 6266 | 79.63% | 82.35% | 18.03% | 98.81% | 0.620 | 0.810 | 0.799 | 0.821 |
| <10.1cm or WAZ <-2 | 6269 | 79.63% | 82.34% | 18.03% | 98.81% | 0.620 | 0.810 | 0.799 | 0.821 |
| <10.2cm or WAZ <-2 | 6277 | 79.63% | 82.31% | 18.00% | 98.81% | 0.619 | 0.810 | 0.799 | 0.820 |
| <10.3cm or WAZ <-2 | 6282 | 79.63% | 82.30% | 17.99% | 98.81% | 0.619 | 0.810 | 0.799 | 0.820 |
| <10.4cm or WAZ <-2 | 6289 | 79.63% | 82.27% | 17.97% | 98.81% | 0.619 | 0.810 | 0.799 | 0.820 |
| <10.5cm or WAZ <-2 | 6290 | 79.63% | 82.27% | 17.97% | 98.81% | 0.619 | 0.810 | 0.799 | 0.820 |
| <10.6cm or WAZ <-2 | 6294 | 79.63% | 82.26% | 17.95% | 98.81% | 0.619 | 0.809 | 0.799 | 0.820 |
| <10.7cm or WAZ <-2 | 6301 | 79.77% | 82.24% | 17.97% | 98.82% | 0.620 | 0.810 | 0.799 | 0.821 |
| <10.8cm or WAZ <-2 | 6304 | 79.77% | 82.23% | 17.96% | 98.81% | 0.620 | 0.810 | 0.799 | 0.821 |
| <10.9cm or WAZ <-2 | 6314 | 79.92% | 82.20% | 17.96% | 98.82% | 0.621 | 0.811 | 0.800 | 0.821 |
| <11cm or WAZ <-2 | 6316 | 79.92% | 82.19% | 17.95% | 98.82% | 0.621 | 0.811 | 0.800 | 0.821 |
| <11.1cm or WAZ <-2 | 6322 | 79.92% | 82.17% | 17.94% | 98.82% | 0.621 | 0.810 | 0.800 | 0.821 |
| <11.2cm or WAZ <-2 | 6338 | 80.13% | 82.13% | 17.94% | 98.83% | 0.623 | 0.811 | 0.801 | 0.822 |
| <11.3cm or WAZ <-2 | 6345 | 80.20% | 82.11% | 17.94% | 98.84% | 0.623 | 0.812 | 0.801 | 0.822 |
| <11.4cm or WAZ <-2 | 6369 | 80.48% | 82.04% | 17.93% | 98.85% | 0.625 | 0.813 | 0.802 | 0.823 |
| <11.5cm or WAZ <-2 | 6383 | 80.62% | 82.00% | 17.92% | 98.86% | 0.626 | 0.813 | 0.803 | 0.824 |
| <11.6cm or WAZ <-2 | 6406 | 80.62% | 81.92% | 17.86% | 98.86% | 0.625 | 0.813 | 0.802 | 0.823 |
| <11.7cm or WAZ <-2 | 6434 | 80.90% | 81.84% | 17.84% | 98.87% | 0.627 | 0.814 | 0.803 | 0.824 |
| <11.8cm or WAZ <-2 | 6449 | 81.04% | 81.79% | 17.83% | 98.88% | 0.628 | 0.814 | 0.804 | 0.825 |
| <11.9cm or WAZ <-2 | 6497 | 81.04% | 81.63% | 17.70% | 98.88% | 0.627 | 0.813 | 0.803 | 0.824 |
| <12cm or WAZ <-2 | 6528 | 81.32% | 81.53% | 17.68% | 98.90% | 0.629 | 0.814 | 0.804 | 0.825 |
| <12.1cm or WAZ <-2 | 6624 | 81.68% | 81.22% | 17.50% | 98.91% | 0.629 | 0.814 | 0.804 | 0.825 |
| <12.2cm or WAZ <-2 | 6778 | 82.10% | 80.71% | 17.19% | 98.93% | 0.628 | 0.814 | 0.804 | 0.824 |
| <12.3cm or WAZ <-2 | 6851 | 82.38% | 80.48% | 17.06% | 98.94% | 0.629 | 0.814 | 0.804 | 0.824 |
| **<12.4cm or WAZ <-2** | **7064** | **83.23%** | **79.79%** | **16.72%** | **98.99%** | **0.630** | **0.815** | **0.805** | **0.825** |
| <12.5cm or WAZ <-2 | 7143 | 83.37% | 79.52% | 16.56% | 98.99% | 0.629 | 0.814 | 0.804 | 0.824 |
| <12.6cm or WAZ <-2 | 7247 | 83.72% | 79.18% | 16.39% | 99.01% | 0.629 | 0.815 | 0.805 | 0.824 |
| <12.7cm or WAZ <-2 | 7462 | 84.50% | 78.48% | 16.07% | 99.05% | 0.630 | 0.815 | 0.805 | 0.825 |
| <12.8cm or WAZ <-2 | 7561 | 84.71% | 78.15% | 15.90% | 99.05% | 0.629 | 0.814 | 0.805 | 0.824 |
| <12.9cm or WAZ <-2 | 7827 | 85.13% | 77.26% | 15.43% | 99.07% | 0.624 | 0.812 | 0.802 | 0.822 |
| <13cm or WAZ <-2 | 7989 | 85.91% | 76.74% | 15.26% | 99.11% | 0.626 | 0.813 | 0.804 | 0.823 |
| <13.1cm or WAZ <-2 | 8351 | 86.54% | 75.52% | 14.70% | 99.14% | 0.621 | 0.810 | 0.801 | 0.820 |
| <13.2cm or WAZ <-2 | 8987 | 87.81% | 73.40% | 13.86% | 99.20% | 0.612 | 0.806 | 0.797 | 0.815 |
| <13.3cm or WAZ <-2 | 9265 | 88.16% | 72.46% | 13.50% | 99.21% | 0.606 | 0.803 | 0.794 | 0.812 |
| <13.4cm or WAZ <-2 | 9808 | 89.08% | 70.64% | 12.89% | 99.25% | 0.597 | 0.799 | 0.790 | 0.807 |
| <13.5cm or WAZ <-2 | 10026 | 89.43% | 69.91% | 12.66% | 99.27% | 0.593 | 0.797 | 0.788 | 0.805 |
| <13.6cm or WAZ <-2 | 10417 | 89.92% | 68.59% | 12.25% | 99.29% | 0.585 | 0.793 | 0.784 | 0.801 |
| <13.7cm or WAZ <-2 | 11036 | 90.49% | 66.49% | 11.63% | 99.31% | 0.570 | 0.785 | 0.777 | 0.793 |
| <13.8cm or WAZ <-2 | 11359 | 90.84% | 65.40% | 11.35% | 99.32% | 0.562 | 0.781 | 0.773 | 0.789 |
| <13.9cm or WAZ <-2 | 12095 | 92.25% | 62.94% | 10.82% | 99.40% | 0.552 | 0.776 | 0.768 | 0.783 |
| <14cm or WAZ <-2 | 12452 | 92.67% | 61.73% | 10.56% | 99.42% | 0.544 | 0.772 | 0.765 | 0.779 |
| <14.1cm or WAZ <-2 | 13189 | 93.23% | 59.23% | 10.03% | 99.45% | 0.525 | 0.762 | 0.755 | 0.769 |
| <14.2cm or WAZ <-2 | 14299 | 94.15% | 55.46% | 9.34% | 99.49% | 0.496 | 0.748 | 0.741 | 0.755 |
| <14.3cm or WAZ <-2 | 14779 | 94.43% | 53.82% | 9.07% | 99.50% | 0.483 | 0.741 | 0.735 | 0.748 |
| <14.4cm or WAZ <-2 | 15645 | 95.14% | 50.88% | 8.63% | 99.54% | 0.460 | 0.730 | 0.724 | 0.736 |
| <14.5cm or WAZ <-2 | 16003 | 95.42% | 49.66% | 8.46% | 99.55% | 0.451 | 0.725 | 0.719 | 0.732 |
| <14.6cm or WAZ <-2 | 16614 | 95.63% | 47.58% | 8.17% | 99.55% | 0.432 | 0.716 | 0.710 | 0.722 |
| <14.7cm or WAZ <-2 | 17561 | 95.98% | 44.34% | 7.76% | 99.56% | 0.403 | 0.702 | 0.696 | 0.707 |
| <14.8cm or WAZ <-2 | 18009 | 96.05% | 42.80% | 7.57% | 99.55% | 0.389 | 0.694 | 0.688 | 0.700 |
| <14.9cm or WAZ <-2 | 18982 | 96.62% | 39.49% | 7.22% | 99.58% | 0.361 | 0.681 | 0.675 | 0.686 |
| <10cm or WAZ <-3 | 1112 | 28.26% | 97.56% | 36.06% | 96.54% | 0.258 | 0.629 | 0.617 | 0.641 |
| <10.1cm or WAZ <-3 | 1115 | 28.26% | 97.55% | 35.96% | 96.54% | 0.258 | 0.629 | 0.617 | 0.641 |
| <10.2cm or WAZ <-3 | 1125 | 28.33% | 97.52% | 35.73% | 96.54% | 0.258 | 0.629 | 0.617 | 0.641 |
| <10.3cm or WAZ <-3 | 1132 | 28.33% | 97.49% | 35.51% | 96.54% | 0.258 | 0.629 | 0.617 | 0.641 |
| <10.4cm or WAZ <-3 | 1142 | 28.40% | 97.46% | 35.29% | 96.54% | 0.259 | 0.629 | 0.618 | 0.641 |
| <10.5cm or WAZ <-3 | 1145 | 28.40% | 97.45% | 35.20% | 96.54% | 0.259 | 0.629 | 0.617 | 0.641 |
| <10.6cm or WAZ <-3 | 1152 | 28.47% | 97.43% | 35.07% | 96.54% | 0.259 | 0.630 | 0.618 | 0.641 |
| <10.7cm or WAZ <-3 | 1162 | 28.61% | 97.40% | 34.94% | 96.55% | 0.260 | 0.630 | 0.618 | 0.642 |
| <10.8cm or WAZ <-3 | 1165 | 28.61% | 97.39% | 34.85% | 96.55% | 0.260 | 0.630 | 0.618 | 0.642 |
| <10.9cm or WAZ <-3 | 1183 | 29.03% | 97.35% | 34.83% | 96.57% | 0.264 | 0.632 | 0.620 | 0.644 |
| <11cm or WAZ <-3 | 1190 | 29.11% | 97.33% | 34.71% | 96.57% | 0.264 | 0.632 | 0.620 | 0.644 |
| <11.1cm or WAZ <-3 | 1208 | 29.18% | 97.27% | 34.27% | 96.57% | 0.264 | 0.632 | 0.620 | 0.644 |
| <11.2cm or WAZ <-3 | 1238 | 29.74% | 97.20% | 34.09% | 96.60% | 0.269 | 0.635 | 0.623 | 0.647 |
| <11.3cm or WAZ <-3 | 1260 | 30.16% | 97.14% | 33.97% | 96.61% | 0.273 | 0.637 | 0.625 | 0.648 |
| <11.4cm or WAZ <-3 | 1305 | 30.66% | 97.01% | 33.33% | 96.63% | 0.277 | 0.638 | 0.626 | 0.650 |
| <11.5cm or WAZ <-3 | 1326 | 30.94% | 96.95% | 33.11% | 96.64% | 0.279 | 0.639 | 0.627 | 0.652 |
| <11.6cm or WAZ <-3 | 1384 | 31.43% | 96.78% | 32.23% | 96.66% | 0.282 | 0.641 | 0.629 | 0.653 |
| <11.7cm or WAZ <-3 | 1445 | 32.42% | 96.62% | 31.83% | 96.70% | 0.290 | 0.645 | 0.633 | 0.657 |
| <11.8cm or WAZ <-3 | 1477 | 32.84% | 96.53% | 31.55% | 96.72% | 0.294 | 0.647 | 0.635 | 0.659 |
| <11.9cm or WAZ <-3 | 1574 | 33.83% | 96.24% | 30.50% | 96.76% | 0.301 | 0.650 | 0.638 | 0.663 |
| <12cm or WAZ <-3 | 1635 | 34.81% | 96.08% | 30.21% | 96.80% | 0.309 | 0.654 | 0.642 | 0.667 |
| <12.1cm or WAZ <-3 | 1799 | 36.22% | 95.58% | 28.57% | 96.85% | 0.318 | 0.659 | 0.646 | 0.672 |
| <12.2cm or WAZ <-3 | 2091 | 38.62% | 94.70% | 26.21% | 96.94% | 0.333 | 0.667 | 0.654 | 0.679 |
| <12.3cm or WAZ <-3 | 2224 | 39.75% | 94.30% | 25.36% | 96.98% | 0.340 | 0.670 | 0.657 | 0.683 |
| <12.4cm or WAZ <-3 | 2551 | 42.14% | 93.29% | 23.44% | 97.06% | 0.354 | 0.677 | 0.664 | 0.690 |
| <12.5cm or WAZ <-3 | 2692 | 43.20% | 92.86% | 22.77% | 97.10% | 0.361 | 0.680 | 0.667 | 0.693 |
| <12.6cm or WAZ <-3 | 2908 | 45.17% | 92.21% | 22.04% | 97.18% | 0.374 | 0.687 | 0.674 | 0.700 |
| <12.7cm or WAZ <-3 | 3268 | 48.48% | 91.13% | 21.05% | 97.32% | 0.396 | 0.698 | 0.685 | 0.711 |
| <12.8cm or WAZ <-3 | 3434 | 50.11% | 90.64% | 20.70% | 97.39% | 0.407 | 0.704 | 0.691 | 0.717 |
| <12.9cm or WAZ <-3 | 3881 | 53.28% | 89.26% | 19.48% | 97.51% | 0.425 | 0.713 | 0.700 | 0.726 |
| <13cm or WAZ <-3 | 4151 | 55.81% | 88.46% | 19.08% | 97.62% | 0.443 | 0.721 | 0.708 | 0.734 |
| <13.1cm or WAZ <-3 | 4694 | 59.34% | 86.76% | 17.94% | 97.77% | 0.461 | 0.731 | 0.718 | 0.743 |
| <13.2cm or WAZ <-3 | 5603 | 64.13% | 83.87% | 16.24% | 97.96% | 0.480 | 0.740 | 0.727 | 0.753 |
| <13.3cm or WAZ <-3 | 6018 | 66.31% | 82.56% | 15.64% | 98.05% | 0.489 | 0.744 | 0.732 | 0.757 |
| <13.4cm or WAZ <-3 | 6792 | 69.70% | 80.06% | 14.56% | 98.19% | 0.498 | 0.749 | 0.737 | 0.761 |
| <13.5cm or WAZ <-3 | 7125 | 71.18% | 78.99% | 14.18% | 98.25% | 0.502 | 0.751 | 0.739 | 0.763 |
| <13.6cm or WAZ <-3 | 7697 | 73.50% | 77.14% | 13.55% | 98.35% | 0.506 | 0.753 | 0.741 | 0.765 |
| <13.7cm or WAZ <-3 | 8567 | 76.89% | 74.31% | 12.73% | 98.51% | 0.512 | 0.756 | 0.745 | 0.767 |
| <13.8cm or WAZ <-3 | 9015 | 78.29% | 72.84% | 12.32% | 98.57% | 0.511 | 0.756 | 0.745 | 0.767 |
| **<13.9cm or WAZ <-3** | **9998** | **81.61%** | **69.63%** | **11.58%** | **98.73%** | **0.512** | **0.756** | **0.746** | **0.767** |
| <14cm or WAZ <-3 | 10483 | 83.09% | 68.03% | 11.25% | 98.80% | 0.511 | 0.756 | 0.745 | 0.766 |
| <14.1cm or WAZ <-3 | 11437 | 84.85% | 64.84% | 10.53% | 98.87% | 0.497 | 0.748 | 0.739 | 0.758 |
| <14.2cm or WAZ <-3 | 12831 | 87.67% | 60.19% | 9.70% | 99.01% | 0.479 | 0.739 | 0.730 | 0.748 |
| <14.3cm or WAZ <-3 | 13424 | 88.37% | 58.18% | 9.34% | 99.03% | 0.466 | 0.733 | 0.724 | 0.742 |
| <14.4cm or WAZ <-3 | 14480 | 90.13% | 54.64% | 8.83% | 99.13% | 0.448 | 0.724 | 0.716 | 0.732 |
| <14.5cm or WAZ <-3 | 14901 | 90.70% | 53.22% | 8.64% | 99.15% | 0.439 | 0.720 | 0.712 | 0.728 |
| <14.6cm or WAZ <-3 | 15639 | 91.68% | 50.73% | 8.32% | 99.21% | 0.424 | 0.712 | 0.704 | 0.720 |
| <14.7cm or WAZ <-3 | 16747 | 92.74% | 46.98% | 7.86% | 99.25% | 0.397 | 0.699 | 0.691 | 0.706 |
| <14.8cm or WAZ <-3 | 17263 | 93.09% | 45.22% | 7.65% | 99.26% | 0.383 | 0.692 | 0.684 | 0.699 |
| <14.9cm or WAZ <-3 | 18389 | 94.50% | 41.42% | 7.29% | 99.36% | 0.359 | 0.680 | 0.673 | 0.686 |

Table 2. Diagnostic performance of MUAC cutoffs in identifying severe wasting

| **MUAC Cut-off** | ***Cases (n)*** | ***Sensitivity*** | ***Specificity*** | ***PPV*** | ***NPV*** | ***Youden*** | ***AUROC*** | ***95% CI*** | |
| --- | --- | --- | --- | --- | --- | --- | --- | --- | --- |
| ***All sample*** |  |  |  |  |  |  |  | **LB** | **UB** |
| <10cm | 55 | 1.94% | 99.84% | 10.91% | 99.01% | 0.018 | 0.509 | 0.501 | 0.517 |
| <10.1cm | 60 | 2.27% | 99.82% | 11.67% | 99.01% | 0.021 | 0.510 | 0.502 | 0.519 |
| <10.2cm | 77 | 3.24% | 99.78% | 12.99% | 99.02% | 0.030 | 0.515 | 0.505 | 0.525 |
| <10.3cm | 84 | 3.56% | 99.76% | 13.10% | 99.02% | 0.033 | 0.517 | 0.506 | 0.527 |
| <10.4cm | 98 | 3.56% | 99.71% | 11.22% | 99.02% | 0.033 | 0.516 | 0.506 | 0.527 |
| <10.5cm | 102 | 3.88% | 99.70% | 11.76% | 99.02% | 0.036 | 0.518 | 0.507 | 0.529 |
| <10.6cm | 112 | 4.85% | 99.68% | 13.39% | 99.03% | 0.045 | 0.523 | 0.511 | 0.535 |
| <10.7cm | 131 | 5.18% | 99.62% | 12.21% | 99.04% | 0.048 | 0.524 | 0.512 | 0.536 |
| <10.8cm | 135 | 5.50% | 99.61% | 12.59% | 99.04% | 0.051 | 0.526 | 0.513 | 0.538 |
| <10.9cm | 160 | 5.83% | 99.53% | 11.25% | 99.04% | 0.054 | 0.527 | 0.514 | 0.540 |
| <11cm | 170 | 6.47% | 99.50% | 11.76% | 99.05% | 0.060 | 0.530 | 0.516 | 0.544 |
| <11.1cm | 196 | 7.44% | 99.43% | 11.73% | 99.06% | 0.069 | 0.534 | 0.520 | 0.549 |
| <11.2cm | 247 | 9.39% | 99.28% | 11.74% | 99.08% | 0.087 | 0.543 | 0.527 | 0.560 |
| <11.3cm | 278 | 10.68% | 99.19% | 11.87% | 99.09% | 0.099 | 0.549 | 0.532 | 0.567 |
| <11.4cm | 339 | 11.97% | 99.00% | 10.91% | 99.10% | 0.110 | 0.555 | 0.537 | 0.573 |
| <11.5cm | 366 | 12.62% | 98.92% | 10.66% | 99.10% | 0.115 | 0.558 | 0.539 | 0.576 |
| <11.6cm | 437 | 14.24% | 98.70% | 10.07% | 99.12% | 0.129 | 0.565 | 0.545 | 0.584 |
| <11.7cm | 519 | 16.18% | 98.45% | 9.63% | 99.14% | 0.146 | 0.573 | 0.553 | 0.594 |
| <11.8cm | 567 | 17.15% | 98.30% | 9.35% | 99.15% | 0.155 | 0.577 | 0.556 | 0.598 |
| <11.9cm | 704 | 19.74% | 97.87% | 8.66% | 99.17% | 0.176 | 0.588 | 0.566 | 0.610 |
| <12cm | 779 | 20.71% | 97.63% | 8.22% | 99.18% | 0.183 | 0.592 | 0.569 | 0.614 |
| <12.1cm | 985 | 22.65% | 96.97% | 7.11% | 99.19% | 0.196 | 0.598 | 0.575 | 0.622 |
| <12.2cm | 1318 | 24.60% | 95.89% | 5.77% | 99.20% | 0.205 | 0.602 | 0.578 | 0.626 |
| <12.3cm | 1470 | 26.21% | 95.40% | 5.51% | 99.22% | 0.216 | 0.608 | 0.583 | 0.633 |
| <12.4cm | 1838 | 29.13% | 94.21% | 4.90% | 99.24% | 0.233 | 0.617 | 0.591 | 0.642 |
| <12.5cm | 1992 | 29.45% | 93.71% | 4.57% | 99.24% | 0.232 | 0.616 | 0.590 | 0.641 |
| <12.6cm | 2233 | 33.01% | 92.95% | 4.57% | 99.27% | 0.260 | 0.630 | 0.603 | 0.656 |
| <12.7cm | 2638 | 37.22% | 91.65% | 4.36% | 99.30% | 0.289 | 0.644 | 0.617 | 0.671 |
| <12.8cm | 2818 | 37.86% | 91.06% | 4.15% | 99.31% | 0.289 | 0.645 | 0.617 | 0.672 |
| <12.9cm | 3310 | 41.75% | 89.47% | 3.90% | 99.34% | 0.312 | 0.656 | 0.629 | 0.684 |
| <13cm | 3611 | 43.37% | 88.49% | 3.71% | 99.35% | 0.319 | 0.659 | 0.632 | 0.687 |
| <13.1cm | 4209 | 45.63% | 86.54% | 3.35% | 99.36% | 0.322 | 0.661 | 0.633 | 0.689 |
| <13.2cm | 5181 | 50.49% | 83.37% | 3.01% | 99.40% | 0.339 | 0.669 | 0.641 | 0.697 |
| <13.3cm | 5620 | 53.07% | 81.94% | 2.92% | 99.42% | 0.350 | 0.675 | 0.647 | 0.703 |
| <13.4cm | 6450 | 56.96% | 79.23% | 2.73% | 99.45% | 0.362 | 0.681 | 0.653 | 0.709 |
| <13.5cm | 6807 | 57.93% | 78.06% | 2.63% | 99.45% | 0.360 | 0.680 | 0.652 | 0.708 |
| **<13.6cm** | **7414** | **61.81%** | **76.09%** | **2.58%** | **99.49%** | **0.379** | **0.690** | **0.662** | **0.717** |
| <13.7cm | 8328 | 64.72% | 73.10% | 2.40% | 99.51% | 0.378 | 0.689 | 0.662 | 0.716 |
| <13.8cm | 8803 | 65.37% | 71.53% | 2.29% | 99.51% | 0.369 | 0.685 | 0.658 | 0.711 |
| <13.9cm | 9807 | 68.28% | 68.24% | 2.15% | 99.53% | 0.365 | 0.683 | 0.656 | 0.709 |
| <14cm | 10305 | 68.61% | 66.59% | 2.06% | 99.52% | 0.352 | 0.676 | 0.650 | 0.702 |
| <14.1cm | 11289 | 70.55% | 63.36% | 1.93% | 99.53% | 0.339 | 0.670 | 0.644 | 0.695 |
| <14.2cm | 12716 | 76.05% | 58.69% | 1.85% | 99.58% | 0.347 | 0.674 | 0.650 | 0.698 |
| <14.3cm | 13319 | 77.67% | 56.71% | 1.80% | 99.60% | 0.344 | 0.672 | 0.648 | 0.695 |
| <14.4cm | 14393 | 80.91% | 53.19% | 1.74% | 99.63% | 0.341 | 0.670 | 0.648 | 0.693 |
| <14.5cm | 14820 | 82.20% | 51.79% | 1.71% | 99.65% | 0.340 | 0.670 | 0.648 | 0.691 |
| <14.6cm | 15567 | 83.17% | 49.33% | 1.65% | 99.65% | 0.325 | 0.662 | 0.641 | 0.684 |
| <14.7cm | 16688 | 85.76% | 45.64% | 1.59% | 99.68% | 0.314 | 0.657 | 0.637 | 0.677 |
| <14.8cm | 17215 | 86.41% | 43.90% | 1.55% | 99.68% | 0.303 | 0.652 | 0.632 | 0.671 |
| <14.9cm | 18349 | 87.70% | 40.16% | 1.48% | 99.69% | 0.279 | 0.639 | 0.621 | 0.658 |
| <10cm or WAZ <-2 | 6266 | 80.26% | 80.08% | 3.96% | 99.75% | 0.603 | 0.802 | 0.779 | 0.824 |
| <10.1cm or WAZ <-2 | 6269 | 80.26% | 80.07% | 3.96% | 99.75% | 0.603 | 0.802 | 0.779 | 0.824 |
| <10.2cm or WAZ <-2 | 6277 | 80.26% | 80.05% | 3.95% | 99.75% | 0.603 | 0.802 | 0.779 | 0.824 |
| <10.3cm or WAZ <-2 | 6282 | 80.26% | 80.03% | 3.95% | 99.75% | 0.603 | 0.801 | 0.779 | 0.824 |
| <10.4cm or WAZ <-2 | 6289 | 80.26% | 80.01% | 3.94% | 99.75% | 0.603 | 0.801 | 0.779 | 0.824 |
| <10.5cm or WAZ <-2 | 6290 | 80.26% | 80.00% | 3.94% | 99.75% | 0.603 | 0.801 | 0.779 | 0.824 |
| <10.6cm or WAZ <-2 | 6294 | 80.26% | 79.99% | 3.94% | 99.75% | 0.602 | 0.801 | 0.779 | 0.824 |
| <10.7cm or WAZ <-2 | 6301 | 80.26% | 79.97% | 3.94% | 99.75% | 0.602 | 0.801 | 0.779 | 0.823 |
| <10.8cm or WAZ <-2 | 6304 | 80.26% | 79.96% | 3.93% | 99.75% | 0.602 | 0.801 | 0.779 | 0.823 |
| <10.9cm or WAZ <-2 | 6314 | 80.26% | 79.92% | 3.93% | 99.75% | 0.602 | 0.801 | 0.779 | 0.823 |
| <11cm or WAZ <-2 | 6316 | 80.26% | 79.92% | 3.93% | 99.75% | 0.602 | 0.801 | 0.779 | 0.823 |
| <11.1cm or WAZ <-2 | 6322 | 80.26% | 79.90% | 3.92% | 99.75% | 0.602 | 0.801 | 0.778 | 0.823 |
| <11.2cm or WAZ <-2 | 6338 | 80.26% | 79.84% | 3.91% | 99.75% | 0.601 | 0.801 | 0.778 | 0.823 |
| <11.3cm or WAZ <-2 | 6345 | 80.26% | 79.82% | 3.91% | 99.75% | 0.601 | 0.800 | 0.778 | 0.823 |
| <11.4cm or WAZ <-2 | 6369 | 80.58% | 79.74% | 3.91% | 99.75% | 0.603 | 0.802 | 0.779 | 0.824 |
| <11.5cm or WAZ <-2 | 6383 | 80.58% | 79.70% | 3.90% | 99.75% | 0.603 | 0.801 | 0.779 | 0.824 |
| **<11.6cm or WAZ <-2** | **6406** | **80.58%** | **79.62%** | **3.89%** | **99.75%** | **0.602** | **0.801** | **0.779** | **0.823** |
| <11.7cm or WAZ <-2 | 6434 | 80.91% | 79.53% | 3.89% | 99.76% | 0.604 | 0.802 | 0.780 | 0.824 |
| <11.8cm or WAZ <-2 | 6449 | 80.91% | 79.48% | 3.88% | 99.75% | 0.604 | 0.802 | 0.780 | 0.824 |
| <11.9cm or WAZ <-2 | 6497 | 80.91% | 79.32% | 3.85% | 99.75% | 0.602 | 0.801 | 0.779 | 0.823 |
| <12cm or WAZ <-2 | 6528 | 80.91% | 79.22% | 3.83% | 99.75% | 0.601 | 0.801 | 0.779 | 0.823 |
| <12.1cm or WAZ <-2 | 6624 | 80.91% | 78.90% | 3.77% | 99.75% | 0.598 | 0.799 | 0.777 | 0.821 |
| <12.2cm or WAZ <-2 | 6778 | 80.91% | 78.39% | 3.69% | 99.75% | 0.593 | 0.796 | 0.774 | 0.819 |
| <12.3cm or WAZ <-2 | 6851 | 80.91% | 78.15% | 3.65% | 99.75% | 0.591 | 0.795 | 0.773 | 0.817 |
| <12.4cm or WAZ <-2 | 7064 | 81.55% | 77.45% | 3.57% | 99.76% | 0.590 | 0.795 | 0.773 | 0.817 |
| <12.5cm or WAZ <-2 | 7143 | 81.55% | 77.19% | 3.53% | 99.76% | 0.587 | 0.794 | 0.772 | 0.816 |
| <12.6cm or WAZ <-2 | 7247 | 81.88% | 76.85% | 3.49% | 99.76% | 0.587 | 0.794 | 0.772 | 0.815 |
| <12.7cm or WAZ <-2 | 7462 | 82.20% | 76.14% | 3.40% | 99.76% | 0.583 | 0.792 | 0.770 | 0.813 |
| <12.8cm or WAZ <-2 | 7561 | 82.52% | 75.82% | 3.37% | 99.76% | 0.583 | 0.792 | 0.770 | 0.813 |
| <12.9cm or WAZ <-2 | 7827 | 82.85% | 74.94% | 3.27% | 99.77% | 0.578 | 0.789 | 0.768 | 0.810 |
| <13cm or WAZ <-2 | 7989 | 83.50% | 74.41% | 3.23% | 99.77% | 0.579 | 0.790 | 0.769 | 0.810 |
| <13.1cm or WAZ <-2 | 8351 | 84.14% | 73.22% | 3.11% | 99.78% | 0.574 | 0.787 | 0.766 | 0.807 |
| <13.2cm or WAZ <-2 | 8987 | 85.11% | 71.13% | 2.93% | 99.79% | 0.562 | 0.781 | 0.761 | 0.801 |
| <13.3cm or WAZ <-2 | 9265 | 85.76% | 70.21% | 2.86% | 99.79% | 0.560 | 0.780 | 0.760 | 0.800 |
| <13.4cm or WAZ <-2 | 9808 | 87.06% | 68.43% | 2.74% | 99.81% | 0.555 | 0.777 | 0.758 | 0.796 |
| <13.5cm or WAZ <-2 | 10026 | 87.06% | 67.71% | 2.68% | 99.80% | 0.548 | 0.774 | 0.755 | 0.793 |
| <13.6cm or WAZ <-2 | 10417 | 88.03% | 66.42% | 2.61% | 99.82% | 0.544 | 0.772 | 0.754 | 0.791 |
| <13.7cm or WAZ <-2 | 11036 | 88.35% | 64.38% | 2.47% | 99.82% | 0.527 | 0.764 | 0.746 | 0.782 |
| <13.8cm or WAZ <-2 | 11359 | 88.35% | 63.31% | 2.40% | 99.81% | 0.517 | 0.758 | 0.740 | 0.776 |
| <13.9cm or WAZ <-2 | 12095 | 89.32% | 60.88% | 2.28% | 99.82% | 0.502 | 0.751 | 0.734 | 0.768 |
| <14cm or WAZ <-2 | 12452 | 89.32% | 59.70% | 2.22% | 99.82% | 0.490 | 0.745 | 0.728 | 0.763 |
| <14.1cm or WAZ <-2 | 13189 | 89.97% | 57.27% | 2.11% | 99.82% | 0.472 | 0.736 | 0.719 | 0.753 |
| <14.2cm or WAZ <-2 | 14299 | 91.26% | 53.61% | 1.97% | 99.83% | 0.449 | 0.724 | 0.708 | 0.740 |
| <14.3cm or WAZ <-2 | 14779 | 91.26% | 52.02% | 1.91% | 99.83% | 0.433 | 0.716 | 0.700 | 0.732 |
| <14.4cm or WAZ <-2 | 15645 | 91.91% | 49.16% | 1.82% | 99.83% | 0.411 | 0.705 | 0.690 | 0.721 |
| <14.5cm or WAZ <-2 | 16003 | 92.23% | 47.98% | 1.78% | 99.83% | 0.402 | 0.701 | 0.686 | 0.716 |
| <14.6cm or WAZ <-2 | 16614 | 92.23% | 45.95% | 1.72% | 99.83% | 0.382 | 0.691 | 0.676 | 0.706 |
| <14.7cm or WAZ <-2 | 17561 | 93.20% | 42.83% | 1.64% | 99.84% | 0.360 | 0.680 | 0.666 | 0.694 |
| <14.8cm or WAZ <-2 | 18009 | 93.53% | 41.35% | 1.60% | 99.84% | 0.349 | 0.674 | 0.660 | 0.688 |
| <14.9cm or WAZ <-2 | 18982 | 93.53% | 38.13% | 1.52% | 99.83% | 0.317 | 0.658 | 0.644 | 0.672 |
| <10cm or WAZ <-3 | 55 | 47.90% | 96.81% | 13.31% | 99.45% | 0.447 | 0.724 | 0.696 | 0.751 |
| <10.1cm or WAZ <-3 | 60 | 47.90% | 96.80% | 13.27% | 99.45% | 0.447 | 0.723 | 0.696 | 0.751 |
| <10.2cm or WAZ <-3 | 77 | 48.22% | 96.77% | 13.24% | 99.46% | 0.450 | 0.725 | 0.697 | 0.753 |
| <10.3cm or WAZ <-3 | 84 | 48.54% | 96.75% | 13.25% | 99.46% | 0.453 | 0.726 | 0.699 | 0.754 |
| <10.4cm or WAZ <-3 | 98 | 48.54% | 96.72% | 13.13% | 99.46% | 0.453 | 0.726 | 0.698 | 0.754 |
| <10.5cm or WAZ <-3 | 102 | 48.54% | 96.71% | 13.10% | 99.46% | 0.453 | 0.726 | 0.698 | 0.754 |
| <10.6cm or WAZ <-3 | 112 | 48.54% | 96.68% | 13.02% | 99.46% | 0.452 | 0.726 | 0.698 | 0.754 |
| <10.7cm or WAZ <-3 | 131 | 48.54% | 96.65% | 12.91% | 99.46% | 0.452 | 0.726 | 0.698 | 0.754 |
| <10.8cm or WAZ <-3 | 135 | 48.54% | 96.64% | 12.88% | 99.46% | 0.452 | 0.726 | 0.698 | 0.754 |
| <10.9cm or WAZ <-3 | 160 | 48.54% | 96.58% | 12.68% | 99.46% | 0.451 | 0.726 | 0.698 | 0.754 |
| <11cm or WAZ <-3 | 170 | 48.54% | 96.56% | 12.61% | 99.46% | 0.451 | 0.726 | 0.698 | 0.753 |
| <11.1cm or WAZ <-3 | 196 | 49.19% | 96.50% | 12.58% | 99.46% | 0.457 | 0.728 | 0.701 | 0.756 |
| <11.2cm or WAZ <-3 | 247 | 49.51% | 96.41% | 12.36% | 99.47% | 0.459 | 0.730 | 0.702 | 0.758 |
| <11.3cm or WAZ <-3 | 278 | 49.84% | 96.34% | 12.22% | 99.47% | 0.462 | 0.731 | 0.703 | 0.759 |
| <11.4cm or WAZ <-3 | 339 | 50.49% | 96.20% | 11.95% | 99.48% | 0.467 | 0.733 | 0.705 | 0.761 |
| <11.5cm or WAZ <-3 | 366 | 50.49% | 96.13% | 11.76% | 99.48% | 0.466 | 0.733 | 0.705 | 0.761 |
| <11.6cm or WAZ <-3 | 437 | 50.81% | 95.94% | 11.34% | 99.48% | 0.467 | 0.734 | 0.706 | 0.762 |
| <11.7cm or WAZ <-3 | 519 | 51.46% | 95.74% | 11.00% | 99.48% | 0.472 | 0.736 | 0.708 | 0.764 |
| <11.8cm or WAZ <-3 | 567 | 51.78% | 95.64% | 10.83% | 99.49% | 0.474 | 0.737 | 0.709 | 0.765 |
| <11.9cm or WAZ <-3 | 704 | 51.78% | 95.32% | 10.17% | 99.49% | 0.471 | 0.735 | 0.708 | 0.763 |
| <12cm or WAZ <-3 | 779 | 51.78% | 95.12% | 9.79% | 99.48% | 0.469 | 0.734 | 0.707 | 0.762 |
| <12.1cm or WAZ <-3 | 985 | 52.10% | 94.58% | 8.95% | 99.48% | 0.467 | 0.733 | 0.705 | 0.761 |
| <12.2cm or WAZ <-3 | 1318 | 52.75% | 93.62% | 7.80% | 99.49% | 0.464 | 0.732 | 0.704 | 0.760 |
| <12.3cm or WAZ <-3 | 1470 | 53.72% | 93.19% | 7.46% | 99.49% | 0.469 | 0.735 | 0.707 | 0.762 |
| <12.4cm or WAZ <-3 | 1838 | 55.66% | 92.13% | 6.74% | 99.51% | 0.478 | 0.739 | 0.711 | 0.767 |
| <12.5cm or WAZ <-3 | 1992 | 55.66% | 91.66% | 6.39% | 99.51% | 0.473 | 0.737 | 0.709 | 0.764 |
| <12.6cm or WAZ <-3 | 2233 | 58.25% | 90.97% | 6.19% | 99.53% | 0.492 | 0.746 | 0.719 | 0.774 |
| <12.7cm or WAZ <-3 | 2638 | 59.55% | 89.79% | 5.63% | 99.54% | 0.493 | 0.747 | 0.719 | 0.774 |
| <12.8cm or WAZ <-3 | 2818 | 59.87% | 89.25% | 5.39% | 99.54% | 0.491 | 0.746 | 0.718 | 0.773 |
| <12.9cm or WAZ <-3 | 3310 | 61.49% | 87.78% | 4.90% | 99.55% | 0.493 | 0.746 | 0.719 | 0.774 |
| <13cm or WAZ <-3 | 3611 | 62.78% | 86.90% | 4.67% | 99.56% | 0.497 | 0.748 | 0.721 | 0.775 |
| <13.1cm or WAZ <-3 | 4209 | 64.72% | 85.13% | 4.26% | 99.58% | 0.499 | 0.749 | 0.722 | 0.776 |
| <13.2cm or WAZ <-3 | 5181 | 67.64% | 82.15% | 3.73% | 99.60% | 0.498 | 0.749 | 0.723 | 0.775 |
| <13.3cm or WAZ <-3 | 5620 | 69.26% | 80.79% | 3.56% | 99.61% | 0.500 | 0.750 | 0.724 | 0.776 |
| **<13.4cm or WAZ <-3** | **6450** | **72.17%** | **78.26%** | **3.28%** | **99.64%** | **0.504** | **0.752** | **0.727** | **0.777** |
| <13.5cm or WAZ <-3 | 6807 | 72.82% | 77.16% | 3.16% | 99.64% | 0.500 | 0.750 | 0.725 | 0.775 |
| <13.6cm or WAZ <-3 | 7414 | 75.08% | 75.29% | 3.01% | 99.66% | 0.504 | 0.752 | 0.728 | 0.776 |
| <13.7cm or WAZ <-3 | 8328 | 76.38% | 72.43% | 2.75% | 99.67% | 0.488 | 0.744 | 0.720 | 0.768 |
| <13.8cm or WAZ <-3 | 8803 | 76.38% | 70.94% | 2.62% | 99.66% | 0.473 | 0.737 | 0.713 | 0.760 |
| <13.9cm or WAZ <-3 | 9807 | 78.32% | 67.71% | 2.42% | 99.67% | 0.460 | 0.730 | 0.707 | 0.753 |
| <14cm or WAZ <-3 | 10305 | 78.32% | 66.10% | 2.31% | 99.67% | 0.444 | 0.722 | 0.699 | 0.745 |
| <14.1cm or WAZ <-3 | 11289 | 79.29% | 62.96% | 2.14% | 99.66% | 0.422 | 0.711 | 0.688 | 0.734 |
| <14.2cm or WAZ <-3 | 12716 | 82.85% | 58.38% | 2.00% | 99.70% | 0.412 | 0.706 | 0.685 | 0.727 |
| <14.3cm or WAZ <-3 | 13319 | 84.14% | 56.43% | 1.94% | 99.71% | 0.406 | 0.703 | 0.682 | 0.723 |
| <14.4cm or WAZ <-3 | 14393 | 86.08% | 52.95% | 1.84% | 99.73% | 0.390 | 0.695 | 0.676 | 0.715 |
| <14.5cm or WAZ <-3 | 14820 | 87.38% | 51.57% | 1.81% | 99.75% | 0.390 | 0.695 | 0.676 | 0.714 |
| <14.6cm or WAZ <-3 | 15567 | 87.70% | 49.13% | 1.73% | 99.74% | 0.368 | 0.684 | 0.666 | 0.703 |
| <14.7cm or WAZ <-3 | 16688 | 89.32% | 45.48% | 1.65% | 99.76% | 0.348 | 0.674 | 0.657 | 0.691 |
| <14.8cm or WAZ <-3 | 17215 | 89.97% | 43.78% | 1.61% | 99.77% | 0.338 | 0.669 | 0.652 | 0.686 |
| <14.9cm or WAZ <-3 | 18349 | 90.61% | 40.06% | 1.52% | 99.76% | 0.307 | 0.653 | 0.637 | 0.670 |
