# Supplementary material for: Determining an optimal case definition using mid-upper arm circumference with or without weight for age to identify childhood wasting in the Philippines
Source: PLoS One. 2024 Dec 27;19(12):e0315253. doi: 10.1371/journal.pone.0315253 (PMC11676897; doi:10.1371/journal.pone.0315253)
Supplement: S2 File — (DOCX) [file pone.0315253.s002.docx]

**Supplementary File 2 (S2)**

***Characteristics of children incorrectly classified (“false positive”) as wasted vis-à-vis Weight for Height z-score***

Table 1 presents the characteristics of false-positive children. For those children falsely labeled as severely wasted and moderately wasted vis-à-vis weight-for-height z-score, the average weight-for-age and height-for-age z-scores range from -2.3 to -2.7, near to the WHZ classification cutoff of Z<-3 and majority of these children are also underweight and stunted. Moreover, most of the children have low dietary diversity scores and are not meeting the minimum acceptable diet.

**Table 1. Characteristics of children incorrectly classified as wasted vis-à-vis Weight for Height z-score**

| **Categories** | | **For severe wasting** | | **For moderate wasting** | |
| --- | --- | --- | --- | --- | --- |
|  |  | **N** | **False Positive** | **n** | **False positive** |
| All sample | | 6,157 | | 5,883 | |
| *Age* |  |  |  |  |  |
|  | 6-23 months | 8924 | 1532 (17.2) | 8924 | 1766 (19.8) |
|  | 24-59 months | 21598 | 4625 (21.4) | 21598 | 4117 (19.1) |
| *Sex* | |  |  |  |  |
|  | Male | 15818 | 3141 (19.9) | 15818 | 2850 (18) |
|  | Female | 14704 | 3016 (20.5) | 14704 | 3033 (20.6) |
| *Underweight Status (weight-for-age)* | |  |  |  |  |
|  | Mean WAZ Z-score | 6157 | -2.5 | 5883 | -2.3 |
|  | Not Underweight | 23841 | 165 (0.7) | 23841 | 770 (3.2) |
|  | Severely Underweight | 1070 | 922 (86.2) | 1070 | 670 (62.6) |
|  | Moderately Underweight | 5169 | 5069 (98.1) | 5169 | 4439 (85.9) |
| *Stunting Status (height-for-age)* | |  |  |  |  |
|  | Mean HFA Z-score | 6157 | -2.7 | 5883 | -2.6 |
|  | Not Stunted | 20680 | 1197 (5.8) | 20680 | 1322 (6.4) |
|  | Severely Stunted | 2721 | 2037 (74.9) | 2721 | 1885 (69.3) |
|  | Moderately Stunted | 7121 | 2923 (41) | 7121 | 2676 (37.6) |
| *Dietary Intake* | |  |  |  |  |
|  | Mean energy intake (calories) | 3115 | 651.2 | 3011 | 637.7 |
|  | Mean intake – Carbohydrates (g) | 3115 | 103 | 3011 | 101.0 |
|  | Mean intake – Protein (g) | 3115 | 21.8 | 3011 | 21.4 |
|  | Mean intake – Fat (g) | 3115 | 17 | 3011 | 16.5 |
| *Food, practices, and services* | |  |  | 1519 |  |
|  | Dietary diversity score - 0 | 232 | 46 (19.8) | 232 | 54 (23.3) |
|  | Dietary diversity score - 1 | 1609 | 318 (19.8) | 1609 | 396 (24.6) |
|  | Dietary diversity score - 2 | 2882 | 487 (16.9) | 2882 | 547 (19) |
|  | Dietary diversity score - 3 | 2305 | 393 (17) | 2305 | 437 (19) |
|  | Dietary diversity score - 4 | 1287 | 206 (16) | 1287 | 242 (18.8) |
|  | Dietary diversity score - 5 | 443 | 60 (13.5) | 443 | 71 (16) |
|  | Dietary diversity score - 6 | 76 | 8 (10.5) | 76 | 8 (10.5) |
|  | Dietary diversity score - 7 | 5 | 1 (20) | 5 | 0 (0) |
|  | MMF (% meeting) | 8031 | 1338 (16.7) | 8031 | 1551 (19.3) |
|  | MAD (% meeting) | 1006 | 1519 (17.2) | 1006 | 194 (19.3) |

Source: Authors' analysis of pooled 2018-2019 Expanded National Nutrition Survey
