# Supplementary material for: Determining an optimal case definition using mid-upper arm circumference with or without weight for age to identify childhood wasting in the Philippines
Source: PLoS One. 2024 Dec 27;19(12):e0315253. doi: 10.1371/journal.pone.0315253 (PMC11676897; doi:10.1371/journal.pone.0315253)
Supplement: S3 File — (DOCX) [file pone.0315253.s003.docx]

**Supplementary File 3 (S3)**

Table 1 shows the comparison of sample characteristics of the full sample (representative) and the final sample with dropped observations due to missing anthropometric data.

**Table 1. Sample characteristics of full representative sample and final sample**

| **Variables** | **Full representative sample** | **Final sample*** |
| --- | --- | --- |
| ***All children*** | **34,092** | **30,522** |
| **Age** |  |  |
| 6-23 months | 10098 (29.6) | 8924 (29.2) |
| 24-59 months | 23994 (70.4) | 21598 (70.8) |
| **Sex** |  |  |
| Male | 17722 (52.0) | 15818 (51.8) |
| Female | 16370 (48.0) | 14704 (48.2) |
| ***Wealth quintile*** |  |  |
| Poorest | 11005 (32.3) | 10227 (33.6) |
| Poor | 7948 (23.4) | 7407 (24.3) |
| Middle | 6099 (17.9) | 5516 (18.1) |
| Richer | 4926 (14.5) | 4236 (13.9) |
| Richest | 4057 (11.9) | 3082 (10.1) |
| ***Type of Residence*** |  |  |
| Rural | 21923 (64.3) | 20108 (65.9) |
| Urban | 12169 (35.7) | 10414 (34.1) |

Note: 3,570 observations were dropped from the dataset because of incomplete anthropometric data
